# Of the Artifical Conversion of Animals to a State of Stony Induration and Indestructiveness, Discovered by Girolamo Segato

**Published:** 1836-10

**Authors:** 


ART. XIV.—*Della artificiale Riduzione a Solidità Lapidea e Inalterabilita degli Animali, scoperta da* GIROLAMO SEGATO. Relazione dell' Avocato GIUSEPPE PELLEGRINI.—*Padova*, 1835. Pp. 44.

*Of the artificial Conversion of Animals to a State of stony Induration and Indestructiveness, discovered by* GIROLAMO SEGATO. By GIUSEPPE PELLEGRINI.

THIS is one of the most extraordinary books we have met with for a long time, in regard to style and composition; but the subject of it is more wonderful still. In the matter of grandiloquence and fustian, we think "the force of nature could no further go" than in the boasting and sonorous periods of Signor Pellegrini; and assuredly, if what he tells us of the doings of the subject of his eulogies, Segato, be in anywise true, art seems also to have reached the limit of her marvels. To reduce any part or the whole of any animal body to a degree of solidity, equal not only to that of marble but that of flint, so as to admit of the most perfect polish, and at the same time to preserve its exact form and colour; to be consequently insusceptible of every destructive process to which animal matter is subject: such is the feat which the historian of this new Gorgonean art not only assures us that Segato can do, but adduces numerous certificates from learned doctors, and anatomists, and chemists, to prove what he has done. Nay, not only can he petrify to this degree, but he can modify his process in such ways as to retain the *flexibility* of the limbs, while the imperishable stoniness remains! Truly, this is something not dreamed of in our philosophy; and, when we couple the astounding facts narrated with the inconceivably bombastic eulogies showered upon the inventor, we can hardly persuade ourselves that the whole is not a piece of humorous invention, and that Girolamo and his petrifactions have not been dug out together from the ruins of some new *Laputa* by some Italian *Swift*. If our reason is startled at the idea of bodies hard as flint and flexible as wax, can we persuade even our imagination that such an address as the following, prefixed as a sort of dedication to this pamphlet, could in truth be written in sober earnest, by a learned and grave professor, to a *bona fide* living man of (unpetrified) flesh and blood?

"To Girolamo Segato—To the new genius of the creative wisdom of Italy, who the mortal remains of man—from top to toe, from flesh to bone, from brain to blood,

—in all the splendour of native colouring petrifies, elasticises, eternises;—To the victor of the imitative arts, amid all his marvels the modestest of men, the prime applauses of the whole earth, *Luigi Muzzi* astonished sends."

And, nevertheless, there seems to us internal evidence in the book that it is no quiz; and, after making ample allowance for the exaggerations of national vanity, the zeal of friendship, and the flights of oratory, we are forced to admit either that a very singular and important discovery has been made, or that the countrymen of Boccaccio are still very accomplished story-tellers.

Signor Segato, we are told, is a great geographer and traveller, and it was in the midst of an excavation left by one of those whirling pillars, or sand-spouts, in the deserts of Ethiopia, that he was led first to conceive the possibility of factitious petrifaction, by the discovery in this place of some human remains exsiccated, *carbonized*, and preserved by the influence of the burning sand. Here "he discovered an entire human body, with flesh and bones entirely carbonized, the one black as charcoal, the other as soot, and both friable. It appeared manifest that the carbonization must be derived from the intense heat of the sand, (incandenza del bollente sabbione,) amid which the body had been buried probably for ages. Now, thought our traveller, if the natural heat of the sand has been able to effect such complete exsiccation and carbonization of animal substances, why may not a more moderate degree of artificial heat produce a less degree of exsiccation and induration, but still sufficient to preserve them from decay? But how and by what means can such a result be obtained? Such was the problem which, from that very instant, Segato set himself to resolve, and which at last he succeeded in solving. His meditations and lucubrations were prolonged for months; but at length a bright thought, like a torch amid darkness, illumined his mind, and gave the clue to his grand discovery." (P. 6.) This is all we are told of the process, which is kept a secret, and certainly we are not likely to derive much insight on the subject from this detail; for few things can be less alike than the black, friable, carbonized bodies of the desert, and the bright and brilliant *human agates* announced as the result of Segato's secret.

The author of this pamphlet, justly calculating on the incredulity of his readers, "having himself doubted of so great a prodigy until convinced by the testimony of his own senses," subjoins certificates from four of the professors at Florence, (Betti, professor of physiology, Zanetti, professor of anatomy, Tozzetti and Gazzeri, professors of chemistry,) in all of which are asserted, from personal inspection, the statements given above. An extract from one of these will suffice.

"Florence, 14 April, 1835.—I, the under-written doctor of medicine, &c., have observed and attentively examined the divers anatomical and anatomico-pathological preparations made by Sig. Girolamo Segato, as likewise those of comparative anatomy, and of various fishes, reptiles, insects, and other animals destined for the collections of natural history; and can assert that, over and above the great anatomical knowledge and practical skill therein by him displayed, the said Segato has found a method hitherto unknown, and exclusively his own invention, whereby the bodies of animals, in whole or in part, are preserved not only in their natural colours, but with the characters which they have acquired in various diseases; and that such parts, moreover, have assumed a degree of hardness which may be termed, in truth, stony, inasmuch as they can be with difficulty scratched, resist the action of the air and moisture as well as the power of the moth, and are consequently absolutely incorruptible and unalterable, &c." (Signed) "ANTONIO TARGIONI TOZZETTI."

(P. 9.)

A similar attestation is subjoined from the Medico-Chirurgical Society of Bologna.

Among the preparations of this kind described in the pamphlet as being in the collection of Segato, are many small quadrupeds, birds, fishes, reptiles, insects, &c., and also many specimens in normal and morbid human anatomy, as hands and feet, the liver, brain, mammæ, &c., all retaining their natural configuration and colour, and only varying in consistence. But what the historian of Segato's discovery (who, it will be recollected, is not a medical man,) seems to regard as his crowning glory is a small table, in which is inlaid a parallelogram composed of 214 pieces of apparently polished stone, of the most splendid colours and as hard as flint, but which ("chi il crederebbe?") are all portions of human membranes, for the most part altered by disease, and assuming various colours according to the nature of the morbid affection. For jaspers and corals we have slices of kidneys, spleens, hearts, livers, &c., some in their natural state, some as altered by disease.

As we have no observations to make on this discovery satisfactory to ourselves, we must leave it to the reflections of our readers and to the testimony of time. We may, however, take the opportunity of making known to our readers another and more intelligible method of preserving dead bodies, also discovered by an Italian, or rather Sicilian physician, Dr. Tranchina, of Palermo. The results of this method were first published in the Sicilian journal *La Cerere*, in May, 1834; but the actual method was first made known and openly practised by the author on the 11th May, 1835, in the military hospital, *Della Trinità*, at Naples. The whole operation consists in the injection of the body, through the carotid artery, with a solution or mixture of two pounds of arsenic in twenty or twenty-four pounds of water, or spirit of wine, coloured with a little red lead or cinnabar; and in the introducing, by means of a trocar, into the abdominal cavity the same solution, in cases where putrefaction had made some progress. In some cases it appears also that the solution is injected into the anus, nostrils, &c. By this process the body is kept fresh, flexible, without smell, and of its natural colour, during more than two months; after which it gradually dries, hardens, becomes of a darker colour, and can be preserved for years. For this discovery, the great importance of which is self-evident, and for its public disclosure, Dr. Tranchina has been presented by the King of Naples with the sum of 3000 ducats, has been raised to the dignity of knight of the order of Francis I., and appointed military surgeon of the second class.*

* Osservatore Medico, No. ix. x. xi., Aprile, Maggio, Giugno, 1835. Omodei Annali, Luglio ed Agosto, 1835.

